# Going for the jugular: Has assessment of the JVP become neglected?

**DOI:** 10.1186/1753-6561-6-S4-O18

**Published:** 2012-07-09

**Authors:** B Artman, AD Hingorani, RJ MacAllister

**Affiliations:** 1 University College London Medical School, UK; 2 University College London Medical School, University College London Hospital, UK

## Introduction

The assessment of the Jugular Venous Pulse (JVP) is arguably one of the most challenging signs to correctly elicit. We present an observational study of the assessment and recording of the JVP in patients presenting as emergencies to University College London Hospital (UCLH). We supplement this with a survey of clinicians undertaken at a medical staff round and a review of textbook recommendations on the measurement of the JVP.

## Methods

A total of 173 adult patients who presented to the A & E department at UCLH over a 4 week period were selected at random for inclusion into the audit. Inclusion criteria included patients who subsequently were admitted to either the Acute Admissions Unit or to the Acute Surgical Ward for further treatment. The notes were assessed for the frequency and method of JVP recording throughout the inpatients' stay. 

## Results

Over 50% of patients in the audit did not have their JVP recorded at any time in their stay in hospital. Only 12% of JVP recordings in the notes quantified the venous pressure. Over 75% of patients treated with fluids or diuretics in A & E did not have their JVP assessed. Only 2 out of the 18 clinical examination textbooks in our survey correctly reproduced the method for examination recommended by Lewis. A survey of clinicians revealed significant uncertainty regarding the correct method for assessment of the jugular venous pulse. 

## Conclusions

The assessment and recording of the JVP in emergency settings is suboptimal. This may be because its measurement is poorly taught in textbooks or at the bedside. Advice on the measurement of the JVP in textbooks should be improved. Alternative approaches such as podcasts and videos demonstrating best practice may be the preferred route for teaching this difficult clinical sign. 

